# Hospital and Institutional News

**Published:** 1919-12-27

**Authors:** 


### HOSPITAL AND INSTITUTIONAL NEWS.

### SCARLET FEVER AND DIPHTHERIA.

The number of cases of scarlet fever notified in England and Wales (including London) during the week which ended on December 13 shows a decline on the number notified during the week before. The cases of diphtheria notified in England and Wales during the same week were slightly higher than in the week before, but the figures indicate that in London a fall in the number of cases had already begun. The suggestion which has been made in some quarters that the present epidemic prevalence of these diseases arises from causes connected with the milk supply is without foundation. There is no difficulty in London as to the supply of diphtheria antitoxin. Inasmuch as scarlet fever and diphtheria are always present to a lesser or a greater extent, the administrative arrangements of the Metropolitan Asylums Board and the London County Council for dealing with cases are kept in working order. The difficulty which has been experienced in the course of the present epidemic in meeting the requirements of all the cases for which hospital accommodation has been sought has arisen partly from the circumstance that the military occupation of the hospitals of the Metropolitan Asylums Board has not yet finally ceased. The Ministry of Health have for some time been taking steps to recover certain of these hospitals for civil use, and the difficulty of finding accommodation for patients is now being progressively overcome. On the more general question, it may be stated that epidemics of scarlet fever occur at intervals of about four or five years, and during inter-epidemic intervals the proportion of susceptible persons undergoes an increase. Scarlet fever was epidemic in England and Wales in the years 1907 and 1914, and the present epidemic differs from those of the above years in being of smaller dimensions, and in the attainment of the maximum prevalence a little later in the season. The periodic or quasi-periodic appearance of epidemics is probably due to the occurrence of a cycle in the life history of the unknown causative organism, and the increase of the non-immune population—causes which are beyond the sphere of administrative control.

### AN INTERESTING MEDICAL DINNER.

The recent Liverpool Medical Institution's dinner was attended among others by Mr. Joseph Conrad, the novelist. He did not speak, nor was he directly alluded to in that part of the speech made by Sir Robert Jones in proposing the health of the guests, though included in it were "the master mariners," with whom perhaps he might have been classed. One of these was "potentate of Labrador," Mr. W. Grenfell, who has helped to form hospitals there, and to look after the needs of deep-sea fishermen. Professor Smith said that the best way to attract medical students to Liverpool is for one hospital to do post-graduation work; a house staff can assist not only the medical student in his training but the medical graduate in his further studies.

### THE EX-SERVICE MAN IN HOSPITAL LIFE.

The Christmas number of *The Ex-Service Man* contains a good deal of information in regard to work and pensions, but will those who have found employment maintain the feeling aroused by a service which they have since left? It is true that there are many organisations for ex-Service men, and that the aim of the paper is to merge these in an Imperial organisation; but these organisations have also to bear the strain of the disruption of the bond which brought them together. On the other hand, unity is strength, and for a long time to come there will be individuals who, without some organisation on which to depend, will be liable to have their past services forgotten, since the effect of the cheap newspaper has been to shorten the public memory to the interval between each edition that comes out. As it is, probably few know of the existence of *The Ex-Service Man*, if only because, to quote the journal, "over 90 per cent of more than three million and a half of demobilised men have actually been reabsorbed into industry." But even five per cent. not thus absorbed is a very large number, and that number will be in a sorry case if its more fortunate comrades fail to remember the bond which once united them, and support, in their prosperity, the organisations and journals allied with them. *The Ex-Service Man* is to be found in institutional life, and because that life encourages comradeship, we ask them if they are doing their duty by their former friends.

### THE NEW "SATURDAY FUND JOURNAL."

With the December number *The Hospital Saturday Fund Journal* appears in a revised form, with larger print, a coloured cover and illustrations. It was once the news-sheet of the Fund: it is now a newspaper. There is a "sketch" by Mr. Pett Ridge, which takes the form of a very short story, and places the importance of visiting day to those who have never been in hospital in a new light. The Dean of Canterbury writes on "The Motive Power of Hospitals." Incidentally he exposes the argument that as men of business members of hospital boards argue not to spend more than they possess. "The fallacy of the argument is that hospitals never know how much they possess. The resources of a 'man of business' are only what he himself possesses. The resources of the hospitals lie in the benevolent . . . and that source is inexhaustible." Mr. Jerome K. Jerome has a ludicrous article on the difficulty of finding people who do not know your stories. Sir William Collins in another article gives reasons why the treatment of the sick and the pursuit of medical training and research are better in voluntary hands, but he does not dispose of the threat, which the fashion of public ignorance at present favours, that lurks in the Ministry of Health and the development of State activities. These mushroom articles pleasantly relieve the ordinary news of the Fund.

### ANTIQUARIAN DISCOVERIES AT GUY'S.

During the past few months excavations have been proceeding underneath where once stood the western portion of the Clinical House at Guy's Hospital, which, built in 1744, is supposed to be the oldest part of the hospital. The object of the work is in connection with a scheme of improvements. The completed excavations showed the following order of strata: the uppermost layer, between four and five feet in thickness, was composed of earth containing a large quantity of broken bricks and cement. In it were found coins belonging mainly to the Georgian era. For three or four feet below this, the next layer contained remains of the Middle Ages. Lower still was a more compact layer, of about five feet in thickness, containing Roman remains. In the deepest layer reached some fragments of metal were unearthed which were so eroded that it cannot be definitely stated whether they were weapons of the Ancient Britons or not. A spiral found is characteristic of Celtic art, and this article is probably of a date prior to, or shortly after, the Roman occupation of England. The Roman coins date from 97 A.D. to the fourth century.

### ST. BART'S "MIRROR."

St. Bartholomew's have followed up their first pictorial essay in advertising by a second and better effort in the shape of a special number of the *Daily Mirror*, dated December 24, 1919. The first attempt of the appeal organisation, while carrying with it a certain success of illusion, failed, for the chief reason, because of dulness. One hospital ward, for instance, is very much like another hospital ward, and pictures of wards given wholesale on several pages do not carry half the conviction of the carefully studied incidents of hospital life so well chosen and photographed in the St. Bart's *Mirror*. Then again, one can have too much portrayal of the chief officers of the big hospital marching across the quadrangle in the company of this or that celebrity. What the hospital does for *you* —*i.e.*, the public—is wisely a strong part of the appeal. There are to-day 2,500 doctors practising in various parts of the country who learned their art of healing at St. Bartholomew's, and the number of nurses who help us in time of trouble in our homes who have been taught what they know at the great hospital is very large indeed.

### RECENT SECRETARIAL APPOINTMENTS.

Mr. Reginald B. Jay having resigned his appointment as secretary, the Board of the Royal Sussex County Hospital, Brighton, decided to advertise this post as vacant, and Mr. L. L. W. Lancaster-Gaye, who was first assistant secretary and then secretary-in-charge for three and a half years, prior to joining H.M. Forces and serving in military hospitals, has been appointed as his successor. Captain Hartley, late acting secretary of the Hospital, who has latterly been doing appeal work there, has resigned this appointment to take up duty at the Royal Victoria and West Hants Hospital as assistant secretary. Prior to the war Captain F. Hartley was assistant at Manchester Children's Hospital, Pendlebury. The Bolingbroke Hospital, Wandsworth Common, are advertising for a secretary at the modest salary of £300 a year.

### TWO INTERESTING CORNISH WAR MEMORIALS.

Among the Cornish War Memorial Schemes is a projected addition to the Redruth Hospital, a scheme which has been realised, so far as ways and means are concerned, almost as soon as announced. To the £8,000 required the Red Cross will give £4,000, and Sir Edward Nicholl, M.P. for Falmouth-Penryn, has promised £4,000, as it were, out of hand. Redruth, by the way, is also commemorating Richard Trevithick, the Cornish inventor of the high-pressure steam engine, who, himself a native of Illogan, had Redruth for his market town. There is in Cornwall a connection between industry and hospitals, and the former sometimes gives its name to an institution, in the case, for instance, of the Miners' and Women's Hospital, Redruth. Sir Edward Nicholl had offered to subscribe to the Trevithick Statue, but since there were two schemes, of which Camborne's was already advanced, he preferred to devote his donation to a general memorial for which an open site has been purchased, and plans for its embellishment prepared. Redruth, therefore, will have no Trevithick Statue, but an addition to its hospital and an open space instead. This is better than duplication; but we are glad that the scheme for a statue has been adopted by another district.

### A SUBJECT FOR INQUIRY.

Arising out of the note in The Hospital on December 20, page 248, regarding the hardship to patients suffering from injuries due to an accident, who are transferred from hospitals to the infirmary of the Lambeth Board of Guardians, Dr. Lionel Baly reported a much more serious case that had occurred. A woman was attended at her home in Southwark by a private practitioner at two o'clock in the morning for confinement. Chloroform was administered, but the operation was not successful, and the patient was taken in a taxicab to St. Thomas's Hospital. Thence she was forwarded to the Lying-in Hospital, York Road, and subsequently taken to Lambeth Infirmary, arriving there at eight o'clock. It was understood that the patient did not leave the taxicab from the time she left home until she reached the infirmary. Here an operation was performed under an anæsthetic, and fortunately the patient was reported to be making satisfactory progress. The Guardians decided to ask for explanations from the hospitals concerned, and to lay the full facts before the Ministry of Health for their consideration.

### WAITING PATIENTS AND POOR-LAW INFIRMARIES.

The house committee of Bagthorpe Infirmary has been instructed by the Nottingham Board of Guardians to admit surgical cases awaiting treatment in the hospital. This policy, which was determined on in March 1914, was interrupted when the military took possession of the infirmary. A surgeon and an *x*-ray operator were appointed. The infirmary, said Mr. T. Banks, who urged the reaffirmation of this policy, is well equipped; no legal disability attaches to any patient, and he hopes, therefore, that the citizens will use the infirmary. Those assisted, who are able to contribute, can make some payment to the Guardians. In a few weeks the infirmary will be once more available. This public-spirited policy should be an example, and is specially valuable in Nottingham, where the voluntary hospital is in difficulties and the shortage of beds is acute.

### THE BRITISH HOSPITAL IN JERUSALEM.

MR. EVELYN CECIL, Secretary-General of the Order of St. John of Jerusalem in England, has received from the Duke of Connaught, the Grand Master of the Order of the Temple of England and Wales, £1,657 for the reinstatement and endowment of the Order's British Ophthalmic Hospital, Jerusalem. It was destroyed when the Turks left the city.

### NIGHTINGALE LETTERS IN THE MARKET.

WE hope that some good friend of St. Thomas's Hospital will observe that ten letters of Florence Nightingale concerning its foundation are now in the market, and may be had for the modest sum of £18. These autograph letters, addressed to Mr. Whitfield, were written between 1858 and 1860, and extend to some thirty-four pages. They discuss plans and suggestions for the hospital, the mortality of nurses, and include Florence Nightingale's reply to Dr. Greenhow's letter in the *Builder* concerning St. Thomas's Hospital. Full details will be found in Mr. Francis Edward's catalogue, number 396, for December, and those interested should communicate with him at 83 High Street, Marylebone. In regard to St. Thomas's, Miss Nightingale wrote, "I think that you have the finest opportunity of building the finest hospital in the world, if you do but take advantage of it." These letters should certainly be possessed by St. Thomas's Hospital Library, and a few friends among the sisters and nurses, failing a single donor, might combine to give their hospital an unexpected and valuable Christmas present. For tradition is kept alive by respecting the records of the past, and few recent hospitals have such a wealth of historical associations as St. Thomas's.

### SHOULD STAMPED ADDRESSED ENVELOPES BE SENT WITH APPEALS?

A CORRESPONDENT writes: "Institutions which, issuing their postal appeals, are in the habit of enclosing a stamped, addressed envelope for reply, are being criticised on the ground that it is waste, and that those who feel called upon to make a contribution will not in any way be influenced by the stamp enclosed." This is often true, but it is the experience of many appeal secretaries that a stamped envelope does bring a larger percentage of replies. A case in point came under my notice:—A country gentleman received an appeal to which he was prepared to respond, but finding he had no stamps, laid it on one side for a future occasion. It was forgotten, and the institution lost its contribution. A society in a Northern city seems to have received much criticism, for its secretary has had attached to the stamped, addressed envelope a small gummed slip which reads: "On the plea of economy we have been criticised for enclosing a stamped, addressed envelope in our appeal. Our experience is, however, that this procedure brings a much larger percentage of replies than when the envelope is not stamped. Therefore, on the plea of economy, we enclose this stamped, addressed envelope." Would it not be possible for an influential deputation representing registered charitable institutions to obtain permission from the Postmaster-General to allow such societies the use of a special envelope, printed under licence from him, and to pay the return postage on all reply envelopes received by them? The face of the envelope could be printed, "Postage on this communication is prepaid by special arrangement with the Postmaster-General." If this idea could be put into operation would it not tend to allay criticism and bring satisfaction to all parties?

### A RE-UNION OF MEDICAL MEN.

A RE-UNION of medical men in the South Midland Division of the British Medical Association is to be held at the Masonic Hall, Northampton, early next month. The South Midland area covers Northants, Beds, and Bucks. About sixty doctors from these counties saw military service. Dr. H. Cropley, Hon. Secretary, 2 Spencer Parade, Northampton, is making most of the necessary arrangements. It may be mentioned that Dr. Cropley has just retired from active participation in the extensive practice he carried on for so many years at East Park Parade, Northampton. Dr. Cropley has for a very long period been medical officer to the Northampton Poor-Law Institution. He is held in wide and deserved esteem.

### IMPROVEMENTS AT BERMONDSEY INFIRMARY.

BERMONDSEY Board of Guardians have appointed Dr. C. Meadows Ryley as specialist for the treatment of diseases of the eye, and Mr. T. B. Layton to treat those affecting the ear, nose and throat. The post of masseuse will be a half-time appointment, but it is hoped that ere long the nurses will all be able to undergo, if they desire, training in massage treatment. The forty-eight hour working week has been introduced for the nursing staff, which, when complete, will number 135. To accommodate the extra nurses required an extension is to be made to the Nurses' Home at a cost of £15,000 to £16,000.

### WALSALL HOSPITAL: NEW SITE.

THE Committee of Walsall Hospital has bought a site of ten acres, near Rushall Church, and once part of the Mellish estate, as a site for a new hospital. A hospital of 300 beds is desired, and, according to an architect's opinion, not more than sixty beds could be added to the present building, which contains ninety. The funds have to be raised before the new site is built upon.

### PRESCRIPTIONS AND PROFITEERING.

A MEDICINE compounded according to a physician's prescription is a medicinal preparation. On the face of it such an assertion of a common truth would not seem to be worth printing, but until the point had been argued before a Divisional Court on November 8, there was a doubt as to whether the truth of the statement was incontrovertible. The important thing now is that a compounded medicine, being in law a medicinal preparation, comes within the schedule of commodities that fall within the purview of the Profiteering Act, and anyone who has grounds for believing that he has been charged an unreasonable price for a bottle or box of prescribed medicine can levy a complaint before a local Profiteering Committee. While there is no cause to disagree with the definition, a case could be made out for the exclusion of prescribed medicines from the schedules under the Act. A man who overcharges for food can be proceeded against as a profiteer, but a restaurant-keeper who serves up food on a plate can charge as much as he thinks he can get for services rendered. Surely the skill required to compound medicine is as worthy of recompense as the skill required to grill a mutton chop. But we are trespassing on the domain of the Profiteering Committee; it is for them to decide whether in particular cases an unreasonable price has been charged for a medicinal preparation.

### THE FATE OF MUNICIPAL MATERNITY HOMES.

Some municipal authorities with maternity homes are finding that women are not unduly anxious to take advantage of them. This may possibly be due to the present high wages, and accordingly some councils are arranging to cater for those who are willing to pay; at the Hull Corporation Health Committee, for example, Dr. Townsend said that the falling off would make it difficult to provide the necessary cases for the pupil midwives. The Committee has decided to admit paying cases when accommodation is available in order to secure the necessary number of cases for pupil midwives at the Home to qualify for the C.M.B. examination.

### THE MATERNITY NURSE AND THE RATES.

It is a good thing from the point of view of public health that general practitioners are able to spare the time to serve on local bodies. In Dorsetshire, for example, there is the little town of Wimborne Minster, with an average of fifty maternity cases per annum, which up to the present has never possessed a nurse who took such cases. When Dr. E. K. Le Fleming brought the matter before the urban council, of which he is a member, the council failed to agree that a maternity nurse is required, in spite of the fact that all the doctors of the town and district supported the proposal. Dr. Le Fleming told the council what other councils who view this matter in a similar light may well take to heart also—namely, that the Ministry of Health is likely in the near future to insist on every council providing sufficient maternity nurses out of the rates for their district, and to provide also an institution for maternity cases among the poor, so that nurses started on voluntary lines now and helped by public bodies will in the end prove a saving to the rates and not a burden. In Dr. Le Fleming's view the days of the voluntarily appointed nurse in country districts is all but over.

### THE NEWQUAY COTTAGE HOSPITAL.

Newquay has decided that its war memorial shall take the form of a cottage hospital. Some £900 is already available, and we hope that Mr. Greenway, the chairman of the hospital scheme, will not have long to wait for the full amount of £5,000 which is required for the building, and the first year's upkeep of £500. If the town rises to the occasion, an anonymous donor is prepared to hand over a cheque for several hundred pounds. There is no difficulty with regard to the land, for Mr. C. Treffry, the lord of the manor, has invited the committee either to choose the site of the hospital, in which case he will convey the freehold to it, or to utilise an existing building on the estate, the reversion of which he will assign them.

### SUPPLY FORTNIGHTS IN DEVON.

A Devonshire vicar has hit upon a scheme for helping the hospital which may commend itself to others. With a view to effecting a substantial reduction in the food bill of the Royal Devon and Exeter Hospital, the Rev. H. B. Burnaby, of Sampford Courtenay, enlisted the help of the farmers and others in his parish, and guaranteed the hospital's supply of dairy produce, vegetables, etc., for a fortnight, as a gift from his parish. It is now proposed to organise similar supply fortnights in other Devonshire areas. It does not require a very big extension of this principle for county hospitals to be provisioned with gifts in this way all the year round.

### NEW PHARMACIST FOR WESTMINSTER HOSPITAL.

Mr. Robert Fouracre has been appointed Chief Pharmacist at the Westminster Hospital, London, to succeed Mr. Alfred E. Tanner, who has now retired after many years in the service of the hospital. Mr. Fouracre holds the "Major" diploma in pharmacy of the Pharmaceutical Society. His hospital experience was obtained at the London Hospital, and latterly he has been pharmacist to the West End Hospital for Nervous Diseases, Welbeck Street.

### THIS WEEK'S DRUG MARKET.

The year closes with some remarkable peace-time drug prices, and there is nothing which seems to indicate that we have yet reached the highest pinnacle of values. The all-round demand is good, and in some cases big. Production has increased substantially, and will no doubt continue to increase, but producers cannot yet keep up with the worldwide demand resulting from the necessity of filling shelves that are still more than half empty. So long as the supply falls short of the demand we may expect to see a continuation of the upward tendency of prices. At the moment the market is quiet, but as soon as stock-taking is over the business of buying and selling will recommence, and with here and there an exception, it is probable that drug prices will be maintained. Under these circumstances there would appear to be little or no advantage in postponing purchases.

